# Oral rehabilitation with dental implants in patients with tooth agenesis: a retrospective study in Helsinki University Hospital, Helsinki, Finland

**DOI:** 10.2340/aos.v84.44181

**Published:** 2025-08-12

**Authors:** Maria Mikkola, Anu Kiukkonen, Nina-Li Avellan, Patricia Stoor, Viivi Mattila, Jussi Oskari Furuholm, Timo Sorsa, Karita Nylund

**Affiliations:** a Department of Oral and Maxillofacial Diseases, Helsinki University Hospital and University of Helsinki, Helsinki, Finland; b Department of Oral and Maxillofacial Diseases, University of Helsinki, Helsinki, Finland; c Division of Periodontology, Department of Dental Medicine, Karolinska Institutet, Stockholm, Sweden

**Keywords:** Tooth agenesis, hypodontia, oligodontia, dental implants, multidisciplinary team

## Abstract

**Objective:**

The aims of this retrospective and descriptive study were to investigate oral rehabilitation with dental implants in tooth agenesis patients treated at Helsinki University Hospital, Finland, between 2015 and 2019, and to analyze implant survival and prosthetic complications within the first 2 years.

**Material and methods:**

This study included 72 patients (42 oligodontia and 30 hypodontia). Data concerning general/oral health and implant treatment modalities were retrieved from hospital records.

**Results:**

Patients were mostly systemically healthy nonsmokers with a mean age of 32.3 years at the time of implant placement. Orthodontic treatment was required for 77.7%, and 31.9% underwent also orthognathic surgery. Patients had 6.6 missing teeth on average and received 2.8 implants each, 205 in total. Tissue augmentation was needed in 64.4% of cases. Screw-retained suprastructures, mainly single crowns (76.4%), were used in 95.5% of cases, all with custom abutments. The mean follow-up time was 25.2 months, with one technical and one mechanical complication. No implants were lost.

**Conclusions:**

With multidisciplinary planning and collaboration, replacement of all teeth in tooth agenesis may not be required. In this study, despite an average of 6.6 missing teeth per patient, only 2.8 implants were placed on average.

## Introduction

Tooth agenesis is a common dental anomaly and is defined as the congenital absence of one or more teeth, excluding third molars [1, 2]. The permanent dentition is much more affected than the primary, in which congenitally missing teeth are reported to be rare [3]. Hypodontia is defined as the congenital absence of one to five permanent teeth, oligodontia as absence of six or more permanent teeth, and anodontia as the total absence of permanent teeth. Most patients with agenesis are missing only one or two teeth. The most frequently missing teeth are the mandibular second premolars (2.9–3.2% of the population), followed by the maxillary lateral incisors (1.6–1.8%), maxillary second premolars (1.4–1.6%), and mandibular incisors (0.2–0.4%) [2, 4, 5]. The prevalence of tooth agenesis varies between 0.2% and 16.2% in different populations, with a higher occurrence in women than men [2, 6]. In Finland, the prevalence of congenitally missing teeth among schoolchildren is 8.0% [7].

Tooth agenesis can result from environmental factors, such as early irradiation of tooth germs, trauma, infection, hormonal and metabolic influences, or genetic factors [8, 9]. It is thus often nonsyndromic and can be associated with oral clefts or part of a syndrome, such as ectodermal dysplasia [10]. Over 300 genes are involved in tooth morphogenesis, which is why hypodontia is also influenced by genetics [11]. Tooth agenesis is also frequently associated with various anomalies in other teeth, for example, delays in development, ectopic eruption, impaction, transposition, retained deciduous teeth, taurodontia, enamel hypoplasia, shortened roots, and reduced tooth dimensions [12, 13].

Patients with congenitally missing teeth may also have dental asymmetry and malocclusion. In cases of severe skeletal deep bite or Class II or III skeletal relationships, a combination of orthodontic treatment and orthognathic surgery may be required before oral rehabilitation [14, 15]. Especially in cases of oligodontia, the congenital absence of tooth follicles often leads to limited alveolar bone dimensions, which may, in turn, affect dentofacial growth [16–18]. Consequently, treatment is challenging and requires careful interdisciplinary planning with orthodontics, prosthodontics, periodontics, endodontics, and oral and maxillofacial surgery [14, 19].

Dental implant (DI) treatment in combination with orthodontic therapy is the most frequent treatment modality for patients with oligodontia [5, 14, 20]. The survival rate of DIs varies from 90% to 97% during 5 to 10 years of follow-up in healthy patients [21–23]. According to Filius et al. [24], the cumulative 10-year survival rate of DIs in hypodontia patients is 89.1%. Survival rate does not reveal whether the prostheses remained free of complications. Prosthetic complications can be considered as technical (relevant to laboratory-fabricated part or its materials) or mechanical (relevant to prefabricated parts, for example, implants and abutment screws) [25].

According to Jung et al. [26], screw loosening in single implant-supported crowns contributes to a cumulative complication incidence of 8.8% over five years, whereas veneering material fractures account for 3.5% during the same period. Technical and mechanical complications cannot be completely avoided, but with prosthetically oriented treatment planning, the risks can be minimized [27].

The aims of this retrospective study were to investigate oral rehabilitation with Dis in tooth agenesis patients treated at Helsinki University Hospital (HUH), Finland, between 2015 and 2019, and to study implant survival rate and prosthetic complications within the first 2 years after loading. The hypothesis was that the overall survival rate of implants placed at HUH is high (> 90%) and that prosthetic complications are rare and minimal.

## Material and methods

### Study design

This retrospective and descriptive study aimed to report implant treatments and short-term technical and mechanical complications among patients with tooth agenesis treated at Helsinki University Hospital, Helsinki, Finland, between 2015 and 2019. The study was performed according to the World Medical Association Declaration of Helsinki, approved by the Helsinki and Uusimaa Hospital Research Committee (HUS/126/2021) and registered in the hospital database.

### Subjects and methods

The patients were identified by searching data concerning DI placements from the patient records of Helsinki University Hospital. From all these patients, those with conditions other than hypodontia or oligodontia were excluded. Demographic data, number and site of missing teeth, treatment modalities (orthodontic, orthognathic surgery, augmentations) with information on prosthetic rehabilitation with DIs, and possible complications were gathered from hospital records.

Thus, altogether 72 patients with agenesis treated with DIs between 2015 and 2019 in the Department of Oral and Maxillofacial Diseases were analyzed in this retrospective study. The patients were referred to Helsinki University Hospital from primary health care and met the criteria (treatment priority index) for society-funded oral rehabilitation with complex treatment needs for functional or esthetic reasons and with a need for special expertise based on multidisciplinary collaboration [28].

The Modified Total Dental Index (mTDI) [29] was used to determine the oral infection burden index assessed from panoramic tomography before implant placement. mTDI considers oral infection burden caused by caries, periodontitis, periapical lesions, and pericoronitis and ranges from 0 to 10. The index was calculated as 1–3 caries lesions, 1 point; 4–7 lesions or no teeth in mandible or maxilla, 2 points; and 8 or more caries lesions or radix or no teeth, 3 points. Alveolar bone loss was recorded as bone loss in the cervical third of a root, 1 point; in the middle third, 2 points; and in the apical third, 3 points. Periapical lesions were calculated as one periapical lesion, 1 point; two periapical lesions, 2 points; and three or more periapical lesions, 3 points. Pericoronitis of partially erupted teeth was recorded as 0 with no pericoronitis or 1 with pericoronitis present. A low mTDI score represented a low infection burden, and a higher score represented a more severe infection burden.

The level of oral hygiene was assessed by a dentist’s clinical assessment of the amount of visible plaque as excellent/good, moderate, or poor.

The follow-up time was recorded from the prosthetic loading until the last appointment in our department. The prosthetic follow-up examination in our department included radiological and clinical assessments, as well as the registration of prosthetic suprastructures, occlusion, the patient’s oral hygiene methods, and visual evaluation of soft tissues and plaque accumulation. Implant survival was recorded as 100% if the implant was not lost [30]. Since the treatment is government funded, the entire treatment process was strictly confined to our hospital, with no involvement from other clinics. Any complications (e.g. biological or prosthetic) that arose during the treatment period were documented and addressed in our department before the treatment was completed.

After the treatment was completed, patients were guided to primary health care and advised to have future oral examinations at primary health care, where clinical controls, general dental care, supportive periodontal care, and most of the prosthetic repairs were performed.

### Data analysis

We used the SPSS statistical software package (SPSS for Macintosh, version 27.0, IBM Corp., Armonk, NY, USA) for data analysis. A Pearson’s chi-square test was used to evaluate differences in the association of categorical variables between the study groups; p-values below 0.05 were considered statistically significant.

## Results

### Demographic data of patients

In this study, 72 patients were included, of which 30 (41.7%) had hypodontia and 42 (58.3%) had oligodontia. Among them, 47 (65.3%) were generally healthy and seven (9.7%) had a genetic disorder or a syndrome: Axin2 mutation (1), Becwith-Wiedemann syndrome (1), Incontinentia pigmenti (3), ectodermal dysplasia (1), and Williams syndrome (1). The most commonly missing teeth were mandibular second premolar (*n* = 104, 21.8%) and maxillary second premolar (*n* = 103, 21.6%), followed by maxillary first premolar (*n* = 59, 12.9%) and maxillary second incisor (*n* = 55, 11.6%), with a mean number of 6.6 missing teeth per patient. The average age upon first implant placement was 32.3 years, and patients were more often male (*n* = 40, 55.6%). At the time of implant placement, 66 (91.7%) patients did not smoke or use snuff. Oral hygiene was good or excellent in 53 (73.6%) patients and oral infection burden, according to mTDI, before implant placement was minor (mTDI 0–1) in 66 (91.7%) patients. More detailed demographic data are provided in Table 1.

**Table 1:** Demographic and oral health data of 72 patients.

			*N* = 72 (%)
**Severity of tooth agenesis**			
Hypodontia			30 (41.7%)
Oligodontia			42 (58.3%)
Anodontia			0
**Missing teeth**			*n* = 476
Maxillary molar			18 (3.8%)
Maxillary second premolar			103 (21.6%)
Maxillary first premolar			59 (12.9%)
Maxillary incisal and canine			69 (14.5%)
Second incisor			55 (11.6%)
Mandibular molar			24 (5%)
Mandibular second premolar			104 (21.8%)
Mandibular first premolar			45 (9.4%)
Mandibular incisal and canine			54 (11.4%)
Mean number of missing teeth/patient			6.6
**Age**			
Range			14–74
Mean			32.3
Male			32.2
Female			32.5
**Gender**			
Male			40 (55.6%)
Female			32 (44.4%)
**Medical history**			
No systemic diseases			47 (65.3%)
Syndrome/genetic disorder			7 (9.7%)
Axin2 mutation			1 (1.4%)
Beckwith-Wiedemann			1 (1.4%)
Incontinentia pigmenti			3 (4.2%)
Ectodermal dysplasia			1 (1.4%)
Williams syndrome			1 (1.4%)
Asthma			5 (7.0%)
Thyroid disease			4 (5.6%)
Rheumatism			3 (4.2%)
Treated cancer			3 (4.2%)
Other disease (e.g. diabetes, inflammatory bowel disease, cardiovascular disease, bleeding disorder)			11 (15.3%)
Radiation therapy (head and neck)			1 (1.4%)
**Smoking**			
No			66 (91.7%)
Yes			4 (5.6%)
Snuff			1 (1.4%)
Electric cigarette			1 (1.4%)
**Oral hygiene**			
Excellent/good			53 (73.6%)
Moderate			12 (16.6%)
Poor			0
Missing data			7 (9.7%)
**Findings in panoramic tomography (mTDI) before implant placement**			
Score Caries			
0	No		55 (76.4%)
1	1–3 lesions		14 (19.4%)
2	4–7 lesions or no teeth in the maxilla or mandible		2 (2.8%)
3	≥ 8 lesions or radix no teeth		1 (1.4%)
**Periodontal**			
0		No alveolar bone loss	64 (88.9%)
1		Alveolar bone loss in the cervical third	5 (6.9%)
2		Alveolar bone loss in the middle third	3 (4.2%)
3		Alveolar bone loss in the apical third	0
**Periapical lesions**			
0		No	68 (94.4%)
1		1 lesion or vertical bone defect	4 (5.6%)
2		2 periapical lesions	0
3		≥3 periapical lesions	0
**Pericoronitis**			
0		No	72 (100%)
1		Yes	0

mTDI: modified total dental index.

### Treatment characteristics

Orthodontic treatment was performed prior to implant and prosthetic treatment. Of 56 (77.8%) patients who underwent orthodontic treatment as part of their prosthetic rehabilitation, 33 (33/56; 58.9%) received only orthodontics and 23 (23/56; 41.1%) also underwent orthognathic surgery. Four (4/56, 7.1%) patients received orthodontic treatment solely for malocclusion, followed by the treatment for tooth agenesis. In all cases, the final prosthodontic treatment plan was determined after the orthodontic treatment had reached the retention phase.

The planning of oral rehabilitation with DIs was prost-hetically oriented. A surgical guide was used in 127 (62%), and two-stage surgery was performed in 183 (89.3%) implant cases. A total of 205 DIs were placed between 2015 and 2019 for oral rehabilitation.

The region of DI placement was maxillary premolar in 66 (32.2%), mandibular premolar in 69 (33.7%), and maxillary incisor and canine in 48 (23.4%) cases. The mean number of DIs placed per patient was 2.8 (ranging from 1 to 10). All 205 DIs were bone-level implants, of which 144 (70.2%) were 4–5 mm wide and 168 (82%) were over 9 mm long. The implant brands used were Straumann 115 (56.1%) with sand-blasted and acid-etched (SLA) and modified (SLActive) surfaces; Astra 76 (37.1%) with titanium grade 4 and acid-etched surface; Xive 11 (5.4%) with titanium grade 2 surface; and Conelog 3 (1.5%) with SLA surface.

Augmentation (hard or soft tissue or both) was needed in 132 (64.4%) implant cases and in 52 (72.2%) patients. Hard tissue augmentation was performed separately before (*n* = 97, 47.3%) and/or during (*n* = 85, 41.5%) the implant placement. Xenografts were used in 123 (60.0%) and autogenous bone grafts in 64 (31.2%) implant cases, of which an iliac crest graft was harvested in 50 (78.1%). With implants placed in maxillary premolar/molar regions, sinus floor augmentation was performed in 48 (68.6%) cases. Hard tissue augmentation was performed more than twice as often in the maxilla than in the mandible (*n* = 86 vs. *n* = 40). Soft tissue augmentation was performed in 44 (21.5%) implant cases, of which 42 (20.5%) were before implantation. Of soft tissue augmentations, xenografts were used in 43 (97.7%) and soft tissue autografts in 3 (6.8%) cases.

One patient (two implants) moved hospital district before implant loading, thus the total number of loaded implants in this study was 203. This has been taken into account in the final results considering suprastructures and follow-up. The prosthetic suprastructure was screw-retained in 194 (95.5%) implant cases and a single crown in 155 (76.4%) cases. Custom abutments were used in all implant-supported crowns and all bridgeworks (*n* = 46 implants, 22.7%) were restored on manufacturers’ bridge abutments. Two implants supported partial denture. In our patient cohort, eight (11.1%) patients received resin-bonded bridges to replace maxillary lateral incisors or mandibular incisors in addition to implant treatment. All surgical and prosthetic components used were original and ordered from the manufacturer to ensure the precise fit of and availability of spare parts.

There were signs of infection prior to implant placement in 20 (15.2%) out of 132 augmentation cases and all of these were seen in patients that underwent additional hard tissue augmentation. Nonresorbable PTFE (Polytetrafluoroethylene) membranes used for hard tissue augmentation before implant placement were related to 18 infections, and 15 out of 32 membranes were removed earlier than planned and patients treated with antibiotics. Three exposed PTFE membranes were left in place and treated with antibiotics and antiseptic mouth rinse. There were four exposed PTFE membranes without signs of an infection, and these were treated with oral antiseptics until removal. Signs of an infection after implant placement were seen in two (1%) cases. There were no significant differences in infection sites between maxilla and mandible, and no implants were lost due to infection.

The follow-up time after prosthetic loading was at least 1 year for 55 (77.5%) patients, and the mean follow-up time was 25.2 months. During the follow-up, none of the implants were lost, and there were only two (1%) prosthetic (technical or mechanical) complications: broken ceramics and one loose abutment screw, both related to a single crown. Treatment characteristics are presented in Table 2. Table 3 provides more detailed information about augmentations, and Table 4 presents data on implants. This retrospective study was based on hospital records, in which no bone loss was registered during the follow-up period.

**Table 2:** Treatment characteristics of 72 patients.

	*N* = 72 (%)
**Orthodontic treatment**	
No	16 (22.2%)
Only orthodontic	33 (45.8%)
Orthognathic surgery	23 (31.9%)
**Imaging before implant placement**	
Cone beam computed tomography	70 (97.2%)
Only panoramic tomography	2 (2.8%)
**Augmentation**	
Yes	52 (72.2%)
No	20 (27.8%)
**Number of implants placed/patient**	
Range	1–10
Mean	2.85
**Number of residual teeth after treatment/patient**	
Range	0–30
Mean	22.3
Number of patients with a minimum of 10 occlusal contacts after treatment	70 (98.6%)*
**Other prosthetic treatments**	
Resin-bonded bridge	8 (11.1%)
Prosthetic crown/veneer	12 (16.7%)
**Follow-up in special dental care unit at HUH**	
No	2 (2.8%)
< 6 months after prosthetics	5 (7.0%)
6–11 months after prosthetics	9 (12.5%)
12–23 months after prosthetics	28 (38.9%)
≥ 24 months after prosthetics	27 (37.5%)
Missing*	1 (1.4%)
Mean follow-up in months	25.2

HUH: Helsinki University Hospital

* One patient moved to another hospital district before implant loading; thus the total number of rehabilitated patients is 71.

**Table 3:** Data of augmentations related to 205 placed implants.

	*N* = 205 (%)
**Augmentation**	
No	73 (35.6%)
Hard tissue	88 (42.9%)
Alveolar ridge preservation	32 (15.6%)
Sinus lift	48 (23.4%)
Graft from iliac crest	50 (24.4%)
Block graft from iliac crest	11 (5.4%)
Block graft from ramus	1 (0.5%)
PTFE membrane used	32 (15.6%)
Soft tissue	0
Both	44 (21.5%)
** Hard tissue**	
** Before implantation**	
No	108 (52.7%)
Autogenous bone	16 (7.8%)
Xenograft	40 (19.5%)
Both	41 (20.0%)
** During**	
No	120 (58.5%)
Autogenous bone	13 (6.3%)
Xenograft	67 (32.7%)
Both	5 (2.4%)
** After**	
No	199 (97.1%)
Autogenous bone	0
Xenograft	5 (2.4%)
Both	1 (0.5%)
** Altogether**	
No	73 (35.6%)
Autogenous bone	9 (4.4%)
Xenograft	68 (33.2%)
Both	55 (26.8%)
** Soft tissue**	
** Before implantation**	
No	163 (79.5%)
Autograft	0
Xenograft	42 (20.5%)
Both	0
** During**	
No	203 (99.0%)
Autograft	1 (0.5%)
Xenograft	1 (0.5%)
** After**	
No	202 (98.6%)
Autograft	2 (1.0%)
Xenograft	1 (0.5%)
** Altogether**	
No	161 (78.5%)
Autograft	1 (0.5%)
Xenograft	41 (20%)
Both	2 (1.0%)
** Infection related to augmentation**	*n* = 132
No infection	106 (80.3%)
No infection, but PFTE membrane exposed	4 (3.0%)
PFTE membrane exposed + antibiotics	3 (2.3%)
PFTE membrane removed + antibiotics	15 (11.4%)
Other signs of infection + antibiotics	2 (1.5%)
PFTE membrane used	32 (24.2%)

PTFE: Polytetrafluoroethylene.

**Table 4:** Data of 205 placed implants.

	*N* = 205 (%)
**Timing of implant placement**	
No need for extraction before implant placement	161 (78.5%)
1–6 months after tooth extraction	14 (6.8%)
6–24 months after tooth extraction	14 (6.8%)
> 24 months after tooth extraction	16 (7.8%)
**Infection after implant placement**	*N* = 205
No	203 (99%)
Yes	2 (1.0%)
**Guided surgery**	
Yes	128 (62.4%)
No	76 (37.1%)
Missing	1 (0.5%)
**Region**	
Maxillary incisal (incisors and canine)	48 (23.4%)
Maxillary premolar	66 (32.2%)
Maxillary molar	4 (2.0%)
Mandibular incisal (incisors and canine)	17 (8.3%)
Mandibular premolar	69 (33.7%)
Mandibular molar	1 (0.5%)
**Implant procedure**	
One-stage	22 (10.7%)
Two-stage	183 (89.3%)
**Implant type**	
Bone level	205 (100%)
Tissue level	0
**Implant diameter**	
< 3 mm	5 (2.4%)
3–4 mm	53 (25.9%)
4–5 mm	144 (70.2%)
5–6 mm	3 (1.5%)
**Implant length**	
< 6 mm	0
6–9 mm	37 (18.0%)
9.1–12 mm	149 (72.7%)
> 12 mm	19 (9.3%)
**Retention to suprastructure**	*n* = 203 *
Screw-retained	194 (95.5%)
Cemented	7 (3.4%)
Locator/Novaloc abutment	2 (1.0%)
**Type of prosthetic structure**	
Single crown	155 (76.4%)
Fixed dental prosthesis	46 (22.7%)
Overdenture	2 (1.0%)
**Signs of prosthetic (technical or mechanical) complications**	
No	201 (99.0%)
Broken ceramics (technical complication)	1 (0.5%)
Loose screw (mechanical complication)	1 (0.5%)
**Survival**	
Yes	203 (100%)
Lost	0

* One patient moved to another hospital district; thus the total number of loaded implants is 203.

## Discussion

The present study describes the oral rehabilitation of 72 patients with tooth agenesis treated with 205 DIs at the Department of Oral and Maxillofacial Diseases Head and Neck Center, Helsinki University Hospital between 2015 and 2019. The extent of the treatment varied significantly depending on the severity of agenesis and the number of implants placed, ranging from 1 to 10 per patient. More than half of the patients had oligodontia (58.3%) and a total of 476 teeth were missing, with an average of 6.6 per patient. In our patient cohort, congenitally missing second molars were not replaced with DIs. However, due to meticulous multidisciplinary treatment planning and orthodontic treatment, the number of inserted implants (2.8 on average per patient) was much less than missing teeth to successfully rehabilitate occlusion. According to the literature, a shortened dental arch is functionally sufficient and can provide long-term occlusal stability [31–34]. Number of patients with a minimum of 10 occlusal contacts after treatment was achieved in 98.6% of cases. Careful planning for achieving reasonable and maintainable outcomes plays an important role since the treatment is funded by the government and expenses need to be taken into consideration.

Tooth agenesis can impair masticatory function and affect oral health, esthetics, and significantly reduce quality of life [35]. Due to the complexity of the treatment, oral rehabilitation planning requires a multidisciplinary team (pedodontist, periodontist, endodontist, orthodontist, oral and maxillofacial surgeon, prosthodontist) to achieve long-term esthetic, functional, and satisfactory results [14, 36]. While conservative prosthodontic treatment has limitations, especially in severe agenesis, implant supported prosthesis has been shown to meet these goals [37–39]. High 5-year survival rates have been reported for implant-supported prostheses, ranging from 97.1% for fixed [40] and 95–100% for removable prosthesis [41]. However, successful oral rehabilitation with DIs often requires both careful patient selection [42–44] and prosthetically oriented treatment planning [27, 45].

A multidisciplinary team in the Department of Oral and Maxillofacial Diseases planned and carried out the treatment. Before implant placement, oral infections were managed, required imaging (cone beam computed tomography, panoramic tomography) was taken, planning was prosthetically driven, and most of the implants (62%) were placed using a surgical guide to optimize positioning. At the time of implant placement, the majority of the patients were generally healthy, nonsmokers, the oral infection burden was minor, and the mean age was slightly over 32 years. All these factors are known to support implant survival [42–44], and in our study, the implant survival rate was 100%.

Prosthetic complications can be considered as technical or mechanical [25]. Technical complications refer to complications of laboratory-fabricated suprasturcture or its materials, such as veneering porcelain chipping or the framework of fixed dental prosthesis [25]. Mechanical complications refer to complications of prefabricated materials such as abutment screws and implants [25].

Common technical/mechanical complications for single implant-retained crowns are fracture or loosening of the abutment/prosthetic screws, loss of retention of cemented crowns, and chipping or fracture of the veneering ceramic [27]. In our study, there was one technical (broken ceramic) and one mechanical (loose abutment screw) complication during the follow-up, which is in line with the literature above. According to Handelsman [45] and Sailer et al. [27], prosthetically driven treatment planning for implant placement reduces the risk for these complications. On the other hand, if such complications occur, Sailer et al. [46] have concluded that screw-retained reconstructions are more easily retrievable, making technical and biological complications easier to manage. In our department, in special dental care, cemented suprastructures are largely avoided, and in our study, almost all restorations were screw retained. In addition, we can speculate that the use of original components in prosthetic suprastructures and custom abutments in every single crown likely contributed to the low incidence of technical and mechanical complications. This assumption is further supported by the literature: with custom-designed abutments, superficial ceramics can receive optimal support [47].

The importance of original components was studied by Fokas et al. [48]. They found significant differences in design and surface contact between the third-party and original abutments. Additionally, in bridgeworks, proper abutment connection ensures passivity and reduces stress on bridge screws. It is also recommended that multiple implant restorations should be done at the abutment level and not the implant level [49]. All bridgeworks in our study were done on bridge abutments and never on fixtures to direct forces away from the rigid endo-osseus part of the structure. No technical or mechanical complications were recorded to bridgework in our study.

Patients with malocclusion often require a combination of orthodontic treatment and orthognathic surgery before implant rehabilitation [14, 15]. In the present study, most of the patients (77.7%) required orthodontic or combined treatment with orthognathic surgery (31.9%) to optimize the space needed for implants and the rehabilitation of the stable occlusion. These findings are in line with previous studies [14, 15]. The congenital absence of tooth follicles can impair alveolar bone growth, and early loss of remaining deciduous teeth may cause alveolar bone atrophy, thus affecting dentofacial growth [15, 16]. Consequently, bone augmentation is often necessary in these patients to enable oral rehabilitation [10, 14]. Hard tissue augmentation was needed in most of the implant cases (64.4%) in our study. A sinus lift was often performed in maxillary premolar/molar regions (23.4% of implants, 37.5% of patients), and in some cases, larger bone grafts from the iliac crest (24.4% of implants, 26% of patients) were harvested for additional vertical and horizontal bone volume. These findings are also consistent with the previous studies of Worsaae et al. [14] and Attia et al. [15].

Before implant placement, soft tissue conditions were carefully considered. According to Lin et al. [50], a lack of adequate keratinized mucosa around DIs is associated with increased plaque accumulation, tissue inflammation, mucosal recession, and attachment loss. However, congenitally missing teeth are often located between other teeth where keratinized mucosa is typically sufficient compared to areas at the end of the dental arch. In our study, soft tissue augmentation was performed in 21.5% of cases, and most were done with xenografts in alveolar ridge preservation procedure aiming to preserve both bone and soft tissue dimensions after tooth (deciduous or permanent) extraction. Alveolar ridge preservation techniques typically involve placing a bone graft material into the socket, which can be further complemented with a barrier membrane or a soft tissue graft to seal the entrance of the socket [51].

In our study, complications related to augmentation were found in one in five augmentation cases. These were mostly associated with hard tissue augmentations when nonresorbable polytetrafluoroethylene (PTFE) barrier membranes were used. As the nonresorbable membranes were removed, there was no infection at the time of implant placement. Our findings are in line with previous literature [52, 53].

Regarding study limitations, patients were managed by several professionals throughout the treatment period, which resulted in variability in the patient records. Due to the retrospective nature of the study, the periodontal status was not systematically recorded, and thus, biological complications could not be reported. In addition, the follow-up time was relatively short to reliably address implant survival rates.

Despite the above-mentioned limitations, we can conclude that patients with tooth agenesis often need orthodontic treatment and hard tissue augmentation before oral rehab-ilitation with implant-supported prosthetic suprastructures. However, through multidisciplinary planning and collaboration, replacement of all missing teeth may not be required. In this study, despite an average of 6.6 missing teeth per patient, only 2.8 implants were placed on average. Short-term prosthetic complications were few; however, longer follow-up is needed to evaluate long-term survival, success, and complications of DIs in patients within this patient group.
